# Perceptual Similarity Can Drive Age-Related Elevation of False Recognition

**DOI:** 10.3389/fpsyg.2019.00743

**Published:** 2019-05-09

**Authors:** Isabelle Boutet, Khalil Dawod, Félix Chiasson, Olivier Brown, Charles Collin

**Affiliations:** School of Psychology, University of Ottawa, Ottawa, ON, Canada

**Keywords:** aging, false recognition errors, face recognition, object recognition, memory

## Abstract

Older adults consistently show elevated rates of false recognition of new items that are related to studied items. This finding has been largely attributed to a greater tendency for older adults to rely on conceptual gist during memory recognition tasks. However, perceptual factors may also be implicated considering that related items are not only conceptually but also perceptually similar. While some findings do suggest that age-related increases in false recognitions can be driven by perceptual factors, little is known about the nature and circumstances under which these factors operate. To address this gap, we measured basic visual ability as well as false recognition for four different image categories (upright faces, inverted faces, chairs, houses) in younger (*n* = 34) and older (*n* = 34) adults. Each image category represented different levels of variability in perceptual similarity and pre-experimental exposure. Perceptual similarity was objectively defined on the basis of the low-level properties of the images. We found evidence that perceptual similarity can contribute to elevated rates of false recognition in older adults. Our results also suggest that declines in basic visual abilities influence elevated false recognition in older adults for perceptually similar but not perceptually dissimilar items. We conclude that both perceptual and conceptual similarity can drive age-related differences in false recognition.

## Introduction

Healthy aging is associated with a decline in recognition memory that is particularly salient for items that share similar characteristics, such as when they are exemplars of the same category (Koutstaal and Schacter, 1997; Koutstaal et al., 1999; Lövdén, 2003; Taconnat and Rémy, 2006; Pidgeon and Morcom, 2014). When study and test items are related, older adults are more likely than younger adults to falsely recognize new items as old. These errors have important implications for cognitive functioning in older adults, where memory deficits are often diagnosed using neuropsychological tests that rely on false recognitions (e.g., Dementia Questionnaire for Persons with Mental Retardation [DMR], Evenhuis, 1996; California Verbal Learning Test [CVLT], Delis et al., 1987; Doors and People, Baddeley et al., 1994; Benton Facial Recognition Test [BFRT], Benton et al., 1994). Different theories exist in the literature to explain age-related increases in false recognition (Rémy et al., 2008), including inefficient pattern separation (Toner et al., 2009) and a bias toward gist processing (Koutstaal and Schacter, 1997; Schacter et al., 1997a; Koutstaal et al., 1999).

Pattern separation is a process whereby stimuli produce distinct neuronal representations at encoding to support later mnemonic discrimination between studied and new items (Wilson et al., 2006; Stark et al., 2013). Aging is thought to lead to inefficient pattern separation, whereby the distinctiveness or pattern separation between neuronal representations is reduced, leading to false recognition of items similar to those presented at study (Wilson et al., 2006; Yassa et al., 2011a,b). Behavioral and imaging studies in humans, as well as studies with animals, support the notion that aging leads to a reduction in pattern separation in the hippocampus and visual cortical areas (Koutstaal et al., 2001b; Chouinard et al., 2008), which may account for increased false recognition in older adults (Wilson et al., 2006; Toner et al., 2009).

Age-related differences in false recognition for related items have also been attributed to a greater reliance on *gist*, and in particular conceptual gist (e.g., Koutstaal and Schacter, 1997; Tun et al., 1998). Gist representations are global in the sense that they capture the essential meaning of the information presented but lack detailed information (Reyna and Brainerd, 1991; Schacter et al., 1997a). Using this framework, it has been proposed that older adults rely more heavily on gist information when performing mnemonic operations and hence are more likely to make errors for related items because their gist traces’ overlap (Koutstaal and Schacter, 1997; Tun et al., 1998; Brainerd and Reyna, 2002). As a result, older adults would be particularly prone to false recognitions when studied and tested items are related because gist information does not differentiate between exemplars of a given category. However, because within-category exemplars share both conceptual and perceptual similarity, perceptual factors may also be at play (Koutstaal, 2003). To examine this question, researchers have measured age differences in false alarms for study and test items that are conceptually and/or perceptually similar (Rankin and Kausler, 1979; Trahan et al., 1986; Koutstaal and Schacter, 1997; Searcy et al., 1999; Koutstaal, 2003; Sommers and Huff, 2003; Pidgeon and Morcom, 2014; Stahl et al., 2016; Burnside et al., 2017; for verbal stimuli see also e.g., Brainerd et al., 1995; Shiffrin et al., 1995; Schacter et al., 1997c; Arndt and Hirshman, 1998; Budson et al., 2003; Ly et al., 2013). For example, Kouststaal et al. (2003) found age-related differences in false recognition for study and test items that were members of the same basic-level category (e.g., candles) and hence were conceptually similar, but not for “abstract” visual images that were perceptually similar. While Pidgeon and Morcom (2014) replicated this finding, they also found that repeated presentations of visually similar abstract shapes heightened false recognitions in older adults. More recently, Burnside et al. (2017) showed that older adults are just as likely to falsely recognize semantically vs. perceptually related new words from studied words.

While these findings suggest that false recognitions can be driven by perceptual similarity, little is known about the nature and circumstances under which perceptual factors contribute to age-related elevation in false recognition. To address this gap, we examined the influence of perceptual factors using two strategies. Our first strategy was to measure the relationship between basic visual abilities and performance on the memory task in all of our participants. Healthy aging is associated with a loss of acuity and contrast sensitivity, losses which arise from optical and cortical factors (e.g., Owsley et al., 1981; Spear, 1993; Wang et al., 2005; Norton et al., 2009; Monge and Madden, 2016). Poor perceptual encoding of study images in older adults may negatively impact processing of perceptual details and heighten errors arising from a reliance on gist (Kouststaal et al., 2003). If this is the case, then basic visual ability may explain some of the variance in false recognition for perceptually similar items.

Our second strategy was to compare age-related differences across four different image categories that differed with respect to perceptual similarity of within-category items and pre-experimental exposure: upright faces, inverted faces, chairs and houses. Faces offer a unique opportunity to examine the factors that drive false recognitions because they are the only stimulus category that humans memorize and recognize at the individual level on a daily basis (Tanaka and Gauthier, 1997). Exposure and attention to individual faces is essential for social interactions, resulting in an increasingly large number of traces of individual faces stored in memory with age (Chaby and Narme, 2009). As such, we assumed that faces had higher levels of pre-experimental exposure and familiarity than the other stimulus categories tested. Moreover, faces may be more susceptible than other stimulus categories to confusions arising from perceptual gist and/or to tax pattern separation because of their high degree of homogeneity (Gauthier et al., 1998; McKone et al., 2006). Like other within-category memory tasks, unfamiliar faces elicit large age-related differences in false recognition alongside preserved true recognition (e.g., Smith and Winograd, 1978; Flicker et al., 1990; Lamont et al., 2005; reviewed by Searcy et al., 1999). However, few studies have directly compared age effects for faces vs. other visual images to examine if they elicit larger elevations in false recognition in older adults (Boutet and Faubert, 2006; Meinhardt-Injac et al., 2014b, Boutet and Meinhardt-Injac, 2018). There is evidence that age-related differences in memory recognition are more pronounced for faces than other matched stimuli (Boutet and Faubert, 2006; Meinhardt-Injac et al., 2014b). However, it is unclear whether these results would generalize to memory recognition because forced-choice testing procedures rely on different underlying mechanisms than old-new testing procedures (Norman and O’Reilly, 2003).

The second image category consisted of inverted faces, which represent a very low level of familiarity and pre-experimental exposure. However, because upright and inverted faces are perceptually identical, they serve as a control for perceptual similarity of the images. If false memories are mainly driven by perceptual similarity, then age differences in false alarms should be comparable in upright and inverted face conditions. The third image category consisted of chairs matched to the faces in terms of perceptual similarity, which was defined as homogeneity between every possible pair of studied and new items with regards to low-level properties of the images such as luminance and contrast (see section “Materials and Methods” for more details). This definition of perceptual similarity offers two advantages: (i) it is objective rather than being based on subjective ratings used elsewhere (Bastin and Van der Linden, 2003; Pidgeon and Morcom, 2014) and (ii) it parallels the response properties of neural networks in the visual cortex (Zeki, 1978) and is therefore is well-matched to the notion of overlapping neural representations. Because impoverished perceptual representations would negatively impact mnemonic discriminations of perceptually similar items, we predicted that these three image categories, which were matched with respect to perceptual similarity, would be similarly, associated to basic visual abilities. The fourth image category consisted of houses which contained more individuating details such trees, windows, and roof peaks, and hence were more perceptually dissimilar than the other image categories. If perceptual similarity drives age-related elevations in false recognition, then our house stimuli should produce the smallest age differences in false recognition.

## Materials and Methods

### Participants

Demographic details for our younger and older adult participants are provided in Table 1. Younger adults were recruited from the University of Ottawa and were awarded class credit for participation. Older adults were recruited from the community using newspaper ads and received a $25 compensation for their time. The University of Ottawa’s Research Ethics Board approved the study.

**Table 1:** Participant characteristics and results for tests of visual ability.

	Younger Adults	Older Adults
	(*n* = 34)	(*n* = 34)
Age	18.9 (1.36)	71.26 (5.91)
Education Level	13.3 (0.65)	15.2 (2.15)
Gender (*N* female)	25	24
FRACT	1.32 (0.34)	0.90 (0.26)
VisTech 1.5 cpd	5.84 (0.81)	5.24 (0.66)
VisTech 3 cpd	6.06 (0.93)	5.58 (0.56)
VisTech 6 cpd	5.97 (0.93)	4.36 (0.74)
VisTech 12 cpd	6.06 (0.91)	3.82 (1.31)
VisTech 18 cpd	5.88 (1.18)	3.21 (1.58)


*Means and (standard deviations) are provided. cpd: Cycles per degree.*

### Materials

#### Freiburg Visual Acuity Test (FrACT) (Bach, 1996)

This test was used to measure high contrast visual acuity. The FrACT uses an adaptive method (Best PEST, Lieberman and Pentland, 1982) to assess a visual threshold, producing acuity ratios ranging from 0.05 (lowest possible score, 20/400 ft. ≈ 6/120 m) to 2.0 (highest possible score, 20/10 ft., ≈ 6/3 m). In the version of the test we used, participants had to identify the orientation of tumbling-E stimuli across 24 trials. Participants completed this test from a viewing distance of 140 cm. One YA and one OA were excluded because their visual acuity ratios were less than 0.5 (i.e., 20/40 ft or 6/12 m).

#### VisTech Near Contrast Sensitivity Test (VCTS 6000)

This test was used to measure contrast sensitivity. The test consists of a small hand-held chart containing five rows and nine columns of circular patches of sinusoidal gratings. The gratings increase in frequency as one descends the rows, and decrease in contrast from left to right across each row. The orientation of the grating varies arbitrarily, being either upright, tilted left 30°, or tilted right 30°. Participants were tasked to indicate the orientation of the gratings (i.e., upright, tilted left, or tilted right) for each patch, from left to right and top to bottom. The last correct indication of each row is considered their contrast threshold for that particular spatial frequency, or row. The contrast threshold for each row is then connected on an assessment chart to create a contrast sensitivity curve, which is compared to a normal sensitivity curve indicated on the assessment chart. This procedure establishes an estimate of the participant’s contrast sensitivity across five spatial frequencies: 1.5, 3, 6, 12, and 18 cycles per degree (cpd).

#### Montreal Cognitive Assessment (MoCA)

See Lawton et al. (2016) for details. This test was used to screen participants for possible mild cognitive impairment. Participants who scored below a 26/30 were to be excluded from the study (no participant was excluded based on this criterion).

#### Old/New Recognition Task

**FIGURE 1:**
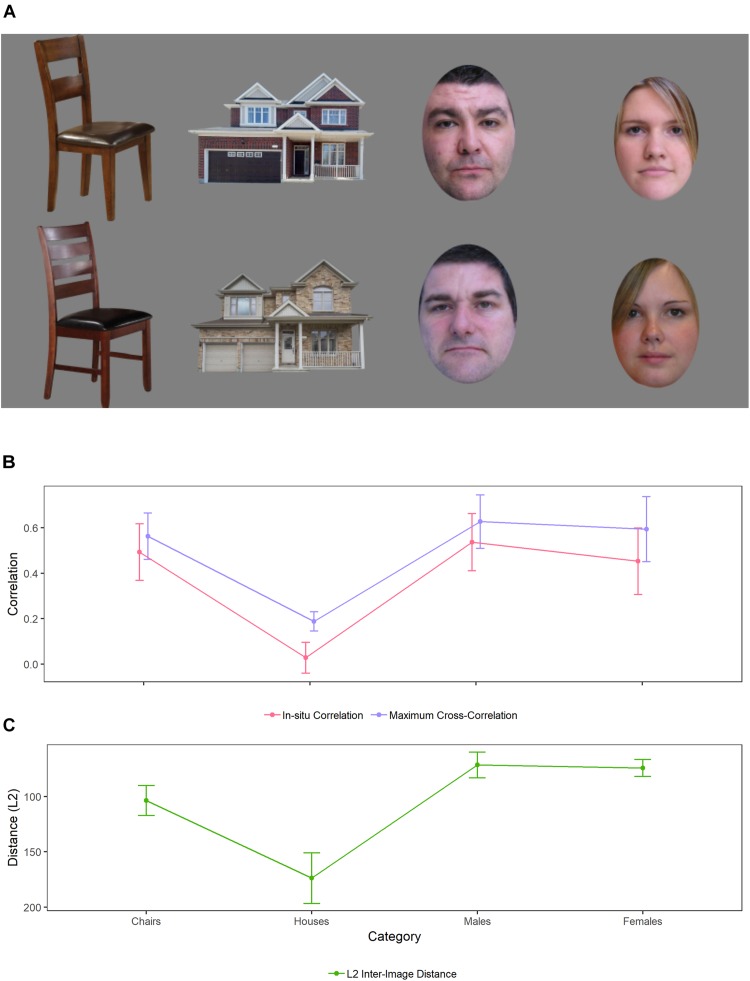
**(A)** illustrates sample target/distractor pairs used for the recognition task. **(B,C)** Illustrates the results of metrics used to calculate physical similarity of the images. Similarity was higher for male faces, female faces and chairs than for houses. Details are provided in the text. Face images were taken from the Glasgow Unfamiliar Face Database (Burton et al., 2010). All individuals whose images appear in the database gave written consent for their images to be used in the context of academic research and publication.

The task was programmed in MATLAB (the mathworks.com) and presented on a 28^′′^ iMac computer. Chair and house stimuli were obtained online. Faces were obtained from the Glasgow Unfamiliar Face Database (Burton et al., 2010). Viewing distance was approximately 65 cm. The images covered approximately 10.5° of visual angle. Chairs were selected on the basis of perceived similarity in an attempt to equate physical homogeneity with upright faces. A co-author, KD, created pairs of chairs to be used as target/distractor on the basis of subjective resemblance of individual images. The set of 20 houses was chosen with the goal of producing similar performance to that obtained with upright faces. However, more variability was present in the original house stimuli than other stimuli because of the presence of distinctive features such as trees that varied in shape, size, and location. We chose to keep these features because recognition of houses in everyday life relies on the use of such information. We measured the homogeneity of basic visual information present in faces, chairs, and houses *via* three objective metrics: *in situ* correlation, cross-correlation, and image difference. These and similar metrics have been used previously to assess image information similarity in ideal observer studies (e.g., Gold et al., 1999; Näsänen, 1999; Sekuler et al., 2004). All metrics yielded similar results, so we focus on the *in situ* correlation here (although all three are presented in Figure 1 for the reader’s reference). *In situ* correlation involves calculating the Pearson product-moment correlation between two paired images’ pixels’ gray levels. This was done for every possible pair of studied and new item across all of the images used for a given image category (for chairs, houses, and male faces and female faces separately). This metric yields a value of 1 for identical images, 0 for completely unrelated ones, and –1 for images with opposite luminance polarity. We then calculated means across every possible pair of studied and new item and 95% CI values for all the indices for all the stimulus categories. Figure 1 shows the results of this analysis as well as examples of the stimulus pairs used in this study. As can be seen in the figure, male faces, female faces, and chairs were equivalent with respect to basic physical homogeneity of target/distractor pairs. In contrast, houses were more heterogeneous.

### Procedure

All participants were tested in the following order: MOCA (for OA only), FrACT, VisTech, recognition task. For the recognition task, four blocks were tested in random order, one for each stimulus category. Each block consisted of the presentation of 10 study targets and 20 test stimuli, in random order. During study, each image was presented for 5 s. During test, each image was presented until the participants indicated via a keyboard press whether the image was old or new. Participants were instructed to take as much time as necessary to provide a correct answer. The test phase immediately followed the study phase. Which image in a pair was to be shown as in the study list vs. in the test list was randomly determined for each participant. At the end of a block, participants were given an optional break, and after the second block, a mandatory 5-minute break. This procedure was chosen on the basis of a pilot study. Our goal was to find an adequate number of learned images and presentation time that would yield neither chance nor ceiling performance in older adults and younger adults across the different image categories tested.

## Results

### Participant Characteristics

For older adults, mean level of cognitive function as measured by the MoCA was 27.80 (*SD* = 1.53). Descriptive statistics for our measures of visual ability can be found in Table 1. VisTech scores were missing from one YA and one OA. When Levene’s test revealed unequal variances, we report results for Welch’s *t*-test instead of Student’s *t*-test. Significant age differences were found for FrACT acuity [*t*(60.56) = 5.71, *p* < 0.001, *d* = 1.41] as well as for contrast sensitivity in all spatial frequencies [1.5 cpd: *t*(64) = 3.36, *p* = 0.001, *d* = 0.84; 3 cpd: *t*(64) = 2.49, *p* = 0.015, *d* = 0.62; 6 cpd: *t*(64) = 7.59, *p* < 0.001, *d* = 1.91; 12 cpd: *t*(56.68) = 8.11, *p* < 0.001, *d* = 2.02; 18 cpd: *t*(59.19) = 7.70, *p* < 0.001, *d* = 1.92].

### Description of Analyses

**FIGURE 2:**
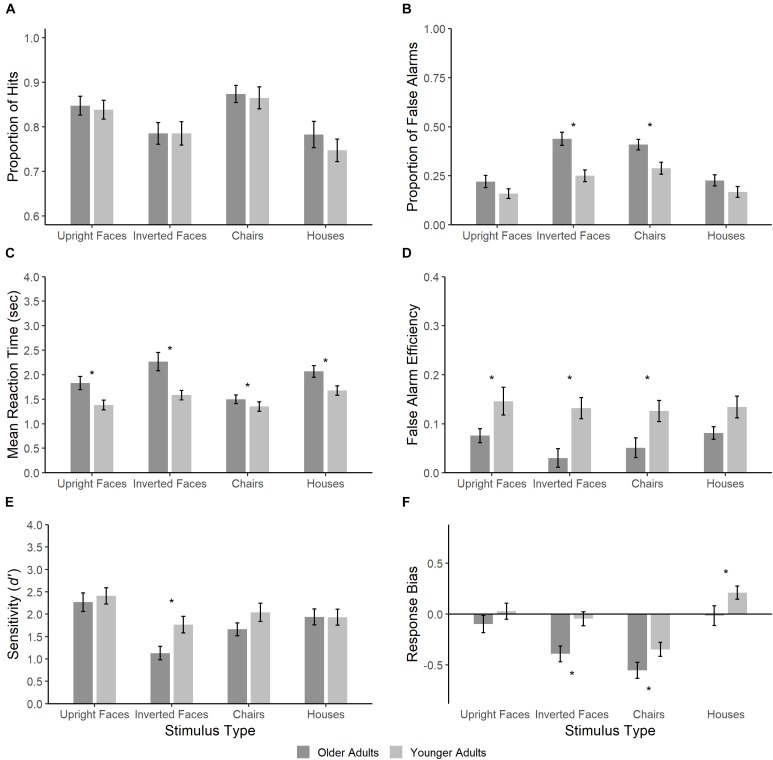
Performance of older and younger adults measured using hits **(A)**, false alarms **(B)**, reaction time **(C)**, false alarms efficiency **(D)**, sensitivity **(E)** and response bias **(F)**. Error bars represent ± 1 standard-error. Asterisks represent significant age differences as revealed by contrast analyses.

Upon visual inspection of our results, we detected differences in speed-accuracy trade-off across the image categories tested. These were most notable for faces, where a lower rate of false alarms was accompanied by longer RTs (see Figure 2). As a result, we added a sixth dependent variable, false alarm efficiency, which was calculated as follows:

$$E_{FA}=\frac{\left(C-A\right)}{R}$$

Where *E_FA_* is the false alarm efficiency, *C* is the chance level of performance (0.5 in this case), *A* is the rate of *FA*s, and *R* is the reaction time in seconds. This score reflects the amount by which false alarms are reduced per second relative to chance-level performance. The higher the efficiency, the better the individual is at using their response time to reduce error. This measure is derived from other studies which employed efficiency measures to combine results obtained on performance and RT (e.g., Townsend and Ashby, 1978; Ramon and Rossion, 2012; Vandierendonck, 2017).

We characterized age-related differences for each image category and dependent variable using planned contrasts (Rosenthal and Rosnow, 1985). We adopted this approach because we stated specific hypotheses regarding age differences in the Introduction and because the means by which aging influences the face inversion effect remains controversial (Chaby et al., 2001; Hildebrandt et al., 2010; Obermeyer et al., 2012; Boutet et al., 2015). Planned contrasts were calculated using the error terms for each relevant interaction derived from the six ANOVAs with Age Group (young adult, older adult) as independent-groups variables and Image Category (upright faces, inverted faces, chairs, houses) as repeated-measures variables computed for each of our dependent variables (Table 2) using SPSS (version 25). While Rosenthal and Rosnow (1985) do not mention the minimum number of comparisons required in order to adjust the alpha level when conducting multiple planned contrasts, the example provided (p. 45) stipulates 8. Therefore, we did not adjust the alpha level when computing contrasts for age differences. Planned contrasts were computed using Excel. Because contrasts are meant to improve statistical power when testing *a priori* hypotheses, we focus on contrasts in our verbal description of the results with the exception of the main effect of Image Category which is reported below because of its relevance for differences in task difficulty.

**Table 2:** Results of 2 × 4 analyses of variance (ANOVA) with Age (young adults vs. older adults) and Image Category (upright faces, inverted faces, chairs, and houses) as variables.

		*df*	*F*	ηp^2^	*p*
False Alarm	Age	1	13.61	0.171	<0.001^*^
	Image Category	3	27.05	0.291	<0.001^*^
	Image Category × Age	3	3.22	0.046	0.024^*^
Hits	Age	1	0.30	0.004	0.588
	Image Category	3	12.38	0.158	<0.001^*^
	Image Category × Age	3	0.31	0.005	0.822
Reaction Time	Age	1	6.92	0.095	0.011^*^
	Image Category	3	26.75	0.288	<0.001^*^
	Image Category × Age	3	2.23	0.033	0.086
*d’*	Age	1	2.34	0.034	0.131
	Image Category	3	13.41	0.169	<0.001^*^
	Image Category × Age	3	2.01	0.030	0.114
Bias	Age	1	9.37	0.124	0.003^*^
	Image Category	3	24.94	0.274	<0.001^*^
	Image Category × Age	3	0.90	0.013	0.444
FA Efficiency	Age	1	22.90	0.258	<0.001^*^
	Image Category	3	1.07	0.016	0.364
	Image Category × Age	3	0.52	0.008	0.667


*n (ANOVA) = 34. ^∗^ significant results.*

To explore the influence of visual ability on behavioral performance, we combined visual acuity and contrast sensitivity for the five spatial frequencies tested using a regression-based method in order to create an overall *z*-score for each participant (Davidson et al., 2018). Because VisTech scores were missing from one YA and one OA, data from these participants was not included for these analyses. This combined visual score was then used to compute bivariate correlations to explore the relationship between visual ability and performance on the memory task.

Assumptions of sphericity and normality were checked using Mauchly’s Test of Sphericity and Shapiro-Wilk tests, respectively. The assumption of normality was met for some image categories and dependent variables but not others. Because exploratory analyses carried on log-transformed values and non-transformed values yielded essentially identical results, we only report analyses of the original data in Tables 3, 4. The assumption of sphericity was met for all measures. The total required sample size estimated with G-power (Faul et al., 2007) for *F*-test repeated-measures, within-between interaction using two groups, four measurements, a power of 0.90 and an effect size of 0.25 was 36. A conservative effect size of *f* = 0.25 was chosen on the basis of previous research where effect sizes ranging from *f* = 0.29 (Lamont et al., 2005) to *f* = 0.87 (Meinhardt-Injac et al., 2017) have been reported.

**Table 3:** Planned contrasts comparing younger to older adults for each image category and dependent variable separately.

	*F*	*p*	Effect size (*r)*
**Upright Faces**			
False Alarm	3.31	0.070	0.130
FA Efficiency	6.41	0.012^*^	0.177
Hits	0.10	0.750	0.023
Reaction Time	17.31	<0.001^*^	0.284
*d’*	0.50	0.481	0.050
Bias	1.77	0.185	0.094
**Inverted Faces**			0.366
FA Efficiency	13.61	<0.001^*^	0.254
Hits	0.00	1.000	0.000
Reaction Time	28.56	<0.001^*^	0.355
*d’*	10.19	0.002^*^	0.221
Bias	13.09	<0.001^*^	0.249
**Chairs**			
False Alarm	12.60	<0.001^*^	0.245
FA Efficiency	7.36	0.007^*^	0.189
Hits	0.10	0.750	0.023
Reaction Time	6.77	0.010^*^	0.182
*d’*	3.63	0.058	0.134
Bias	4.68	0.032^*^	0.152
**Houses**			
False Alarm	3.00	0.085	0.122
FA Efficiency	3.67	0.057	0.135
Hits	1.63	0.204	0.090
Reaction Time	36.10	<0.001^*^	0.393
*d’*	0.00	0.967	0.003
Bias	5.53	0.020^*^	0.165


*n (ANOVA) = 34. ^∗^significant results.*

**Table 4:** Pearson Correlation coefficients between combined vision score and performance for each image category.

	Upright Faces	Inverted Faces	Chairs	Houses
False Alarm	-0.32^**^	-0.35^**^	-0.38^**^	0.02
FA Efficiency	-0.30^*^	-0.18	-0.30^*^	0.03
Reaction Time	-0.16	-0.16	-0.08	-0.24
*d’*	0.10	0.33^**^	0.24	-0.10


*^∗∗^*p* = < 0.01, ^∗^*p* = < 0.05.*

### Performance Differences Across Image Categories

The main effect of Image Category was significant for false alarms. *Post hoc* paired samples *t*-tests revealed that the following comparisons were significantly different: upright faces vs. inverted faces [*t*(67) = -6.05, *p* < 0.001], chairs vs. houses [*t*(67) = 6.19, *p* < 0.001], upright faces vs. chairs [*t*(67) = -6.77 *p* < 0.001], inverted faces vs. house [*t*(67) = 6.12, *p* < 0.001]. In all these comparisons, chair and inverted faces always had the highest number of false recognitions. The main effect of Image Category was also significant for hits. *Post hoc* paired samples *t*-tests revealed that the following comparisons were significantly different: upright faces vs. inverted faces [*t*(67) = 3.04, *p* = 0.003], chairs vs. houses [*t*(67) = 5.13, *p* < 0.001], upright faces vs. houses [*t*(67) = 3.74, *p* < 0.001], inverted faces vs. chairs [*t*(67) = -5.02, *p* < 0.001]. For all these comparisons, upright faces and chairs showed the highest amount of hits. Similarly, the main effect of Image Category was significant for reaction time. Further paired samples *t*-tests revealed significant differences in the following comparisons: upright faces vs. inverted faces [*t*(67) = -5.73, *p* < 0.001], chairs vs. houses [*t*(67) = -7.17, *p* < 0.001], upright faces vs. houses [*t*(67) = -5.14, *p* < 0.001], inverted faces vs. chairs [*t*(67) = 7.09, *p* < 0.001]. For all these comparisons, inverted faces and houses showed the highest mean reaction time. Sensitivity (*d’*) was also significantly different between Image Category. *Post hoc* paired samples *t*-tests revealed significant differences between the following comparisons: upright faces vs. inverted faces [*t*(67) = 6.31, *p* < 0.001], upright faces vs. houses [*t*(67) = 2.83, *p* = 0.006], inverted faces vs. chairs [*t*(67) = -3.11, *p* = 0.003], upright faces vs. chairs [*t*(67) = 3.10, *p* = 0.003], inverted faces vs. houses [*t*(67) = -3.59, *p* = 0.001]. Finally, the main effect of Image Category was significant for response bias. *t*-tests revealed significant differences between the following comparisons: upright faces vs. inverted faces [*t*(67) = 2.76, *p* = 0.007], chairs vs. houses [*t*(67) = -7.80, *p* < 0.001], inverted faces vs. chairs [*t*(67) = 3.82, *p* < 0.001], upright faces vs. chairs [*t*(67) = 5.87, *p* < 0.001], inverted-houses [*t*(67) = -5.32, *p* < 0.001]. Overall, participants responded more liberally when shown the chair stimuli as opposed to other image categories. While not as strong, inverted faces also had a more liberal response bias.

Overall, these results suggest that task difficulty was not equivalent across conditions. Focusing on our main dependent variable of interest, false alarms, we found that inverted faces and chairs produced a higher number of false alarms than upright faces and houses. However, it is important to note that the main effect of Image Category was not significant for false alarm efficiency. This finding corroborates our suspicion that different conditions produced different speed-accuracy trade-offs in false alarms and underscores the importance of including the false alarm efficiency measure in our subsequent analyses.

### Age Differences

Table 3 describes the results of contrast analyses comparing the two age groups for each stimulus category and dependent variable.

#### Number of False Alarms

Significant moderate age differences were found for inverted faces and chairs with older adults committing more false alarms than younger adults. A tendency toward age effects was also found for upright faces and houses, albeit not statistically significant.

#### False Alarm Efficiency

We turn now to false alarm efficiency, which provides a more meaningful measure of false recognition because of differences in speed-accuracy trade-off across conditions (e.g., Townsend and Ashby, 1978; Ramon and Rossion, 2012; Vandierendonck, 2017). Age differences were present and of comparable magnitude for upright faces, inverted faces and chairs. For houses, the significance value was slightly above the alpha level.

#### Hits

There were no significant age differences for number of hits across all four object categories.

#### Mean Reaction Time

Age differences were significant and of moderate magnitude for all stimulus categories.

#### Sensitivity (*d’*)

Age differences were not significant for *d’* for upright faces and houses but significant for inverted faces. For chairs, there was a tendency for sensitivity to be lower in older adults than in young adults.

#### Response Bias (c)

While age differences in response bias were not significant for faces, they were for inverted faces, chairs, and houses. These findings are characterized by a more liberal bias in older adults for all three object categories.

### Correlations Between Performance and Visual Ability

We computed Pearson Correlations (Table 4) to explore the relation between false recognition and visual ability. The combined measure of visual ability was strongly and significantly correlated with the number of false alarms for upright faces, inverted faces, and chairs but not houses. The measure of visual ability was negatively and significantly correlated with false alarm efficiency for upright faces and chairs, but not inverted faces and houses.

## Discussion

We investigated the contribution of perceptual factors to age-related differences in false recognition by comparing performance of younger and older adults on within-category recognition of upright faces, inverted faces, chairs, and houses. Upright faces, inverted faces and chairs were comparable in terms of their physical similarity. Houses were perceptually more dissimilar than the other three categories. Perceptual similarity was operationally defined as the similarity in the low-level physical properties of our stimuli (contrast, luminance) using an objective measure that parallels the response profile of neurons in the visual cortex (Zeki, 1978). Because faces play an important role in social interactions, we assumed that pre-experimental exposure varied incrementally from upright faces, to chairs and houses, to inverted faces. We also examined the influence of perceptual factors by measuring the relationship between basic visual abilities and false recognition. While several measures of performance were included, we focus our discussion on dependent variables that reflect false recognition errors, namely false alarms and false alarm efficiency.

Upright faces elicited almost significant and significant age-related differences for number of false alarms and false alarm efficiency, respectively. This finding corroborates previous evidence that older adults have a greater propensity to endorse new faces as old (false alarms) and yet are comparable to younger adults in their ability to recognize studied faces as old (hits/true recognition) (reviewed by Searcy et al., 1999). Age-related differences were comparable for inverted faces and chairs. For these three image categories, false alarm and false alarm efficiency significantly correlated with our measure of basic visual ability. Taken together, these results support the notion that perceptual similarity can drive age-related elevation in false recognition of related items (Koutstaal and Schacter, 1997; Tun et al., 1998; Koutstaal, 2003; Pidgeon and Morcom, 2014). Age-related effects were weaker and non-significant for house stimuli, which were perceptually more dissimilar than the other categories tested. Moreover, false recognitions on this task did not correlate with visual ability. There is evidence that stimuli that are rich in distinctive information are more likely to elicit conceptual encoding (e.g., Hunt, 2003; Mccabe et al., 2004; Taconnat et al., 2006). It is possible that false recognition of house stimuli was not influenced by perceptual factors because this condition elicited conceptual encoding.

Our findings add to the increasingly large body of evidence suggesting that age-related low-level perceptual decline can influence higher level perceptual and cognitive tasks (e.g., Dupuis et al., 2014; Monge and Madden, 2016). However, with the exception of faces, very few studies have examined false recognitions for individual recognition of perceptually homogeneous stimuli. The results of the current study do not support the contention that unfamiliar face recognition is particularly vulnerable to perceptual degradation (Owsley et al., 1981; Cronin-Golomb et al., 2007; Norton et al., 2009; Boutet and Meinhardt-Injac, 2018; Boutet et al., 2019). In the context of faces, it has been proposed that age-related differences in face recognition arise from older adults relying more heavily on familiarity-based responding than younger adults. In our study, older adults were more likely to adopt a liberal response bias, which supports familiarity-based accounts of false memory (e.g., Trahan et al., 1986; Schacter et al., 1997b; Searcy et al., 1999; Bastin and Van der Linden, 2003). Including remember/know measures would provide a more direct evaluation of this hypothesis. We were surprised to find that upright faces did not elicit significant age differences in response bias, which is in contradiction with past research (Meinhardt-Injac et al., 2014a, 2017; Boutet and Meinhardt-Injac, 2018). One possibility is that ours is a spurious result arising from the high proportion of false alarms and hits elicited by upright faces in our study, although this was not found for houses, which elicited similarly, high levels of performance^1^. While we are not aware of such measures, perhaps calculating bias using both false alarm and RT would have revealed significant age effects for faces.

Our findings are consistent with more recent investigations in suggesting that perceptual similarity can drive false recognition of pictorial images (Kouststaal et al., 2003; Yassa et al., 2011a; Pidgeon and Morcom, 2014). In contrast, there is a long tradition of research using verbal stimuli which underscores the role of conceptual similarity in age-related differences in false recognition (Taconnat and Rémy, 2006) and we interpret our findings with house stimuli in the same direction. Contrast analyses, which afford more power when a small number of pairwise comparisons are needed to answer focal research questions (Rosenthal and Rosnow, 1985), revealed that houses elicited weaker and non-significant age-related effects. We note, however, that the omnibus interaction between Age and Image Category was not significant (Table 2). House stimuli may have elicited conceptual encoding because of their familiarity and because they contained more distinguishing visual information than the other categories. Parallel findings have been reported with images containing distinctive features (reviewed by Hunt, 2003; Mccabe et al., 2004; Thomas and Sommers, 2005; Taconnat and Rémy, 2006) and with abstract pictures that had been repeatedly presented in the experiment (Pidgeon and Morcom, 2014). Our study differs from previous efforts in that we compared familiar image categories rather than abstract images (Koutstaal, 2003; Weinstein and Shanks, 2008; Pidgeon and Morcom, 2014).

As a whole, we interpret our results as supporting the idea that both perceptual and conceptual similarity can drive age-related differences in false recognition depending on the circumstances under which they arise. Differences in visual ability of the participants, perceptual similarity and means of measuring it, presence of perceptual details and amount and nature of pre-experimental exposure may all explain inconsistencies reported in the literature (e.g., see Kouststaal et al., 2003 vs. Pidgeon and Morcom, 2014). In the context of gist theories, our results add to the increasing body of evidence suggesting that older adults are more likely to rely on gist recollection than younger adults when making mnemonic discriminations among items that are related (Koutstaal and Schacter, 1997; Schacter et al., 1997b; Tun et al., 1998; Koutstaal et al., 2001a). Our study adds to this body of evidence by showing that both conceptual and perceptual similarity can drive gist-based recollection. In the context of pattern separation, Ly et al. (2013) have suggested that perceptual, but not conceptual, mnemonic discriminations are negatively affected by inefficient pattern separation. In contrast, Pidgeon and Morcom (2014, 2016) have suggested that studied and test items that are both perceptually and conceptually similar are more likely to elicit elevated false recognition, perhaps because an overlap in perceptual neural representations cannot be compensated with a mnemonic discrimination of conceptual representations and vice-versa. Additional research is needed to clarify the relative importance and potential interaction between perceptual vs. conceptual similarity in driving false recognitions, taxing pattern separation, and gist processing.

This study, along with others on false recognition of pictorial images (Trahan et al., 1986; Koutstaal and Schacter, 1997; Searcy et al., 1999; Kouststaal et al., 2003; Pidgeon and Morcom, 2014; Pidgeon and Morcom, 2016), has important implications for living activities and assessment of older individuals. For example, false recognition of strangers as a result of poor vision may lead to social misunderstandings with known negative consequences on psychological and physical health (Berkman and Syme, 1979; Berkman et al., 2000; Cohen, 2004; Umberson et al., 2006). Our results also imply that identifications may be particularly unreliable for witnesses with reduced vision, which represents 17.4% of the population aged 65 and over (Schiller and Peregoy, 2012). It is important to note here that all our participants had normal-to-corrected vision and had underwent an eye exam within the last year and yet showed significant reduction in basic visual ability as compared to the younger adults (see REFS for similar findings in Neargarder et al., 2003; Cronin-Golomb et al., 2007; Rousselet et al., 2008). The use of corrective lenses may therefore not be a safeguard against recognition errors, albeit not wearing corrective lenses, which is common in this population (Wang et al., 1994), would make matters even worse. It should be noted, however, that age-related differences in sensitivity (*d’*) were not modulated by visual ability for faces, which is at odds with models of signal detection theory (e.g., Macmillan and Creelman, 2005). Indeed, if perceptual degradation leads to a weakened signal, then we would have expected sensitivity to be related to visual ability for these images. Sensitivity is not always reported in studies on false recognition, making it difficult to draw inferences from the literature for this finding. Finally, our results suggest that the interpretation of neuropsychological tests that use faces and/or other within-category exemplars to measure the integrity of memory systems may be confounded by degraded visual ability [e.g., DMR (Evenhuis, 1996), CVLT (Delis et al., 1987), Doors and People (Baddeley et al., 1994), and BFRT (Benton et al., 1994)]. Accordingly, Davidson et al. (2018) have shown that performance on the Mnemonic Similarity Test (MST) is influenced by age-related perceptual decline.

### Limitations

While we choose to employ familiar images that varied naturally with respect to similarity and pre-experimental exposure to enhance the external validity of our study, this approach prevented us from systematically varying these factors. For example, we did not include explicit measures of conceptual encoding in this study but instead inferred that houses elicited more conceptual processing because (i) the relationship between visual ability and false recognitions of houses was not significant, (ii) houses were more objectively dissimilar in terms of the information content of the images, and (iii) houses contained more distinctive details, which are known to encourage conceptual processing (Hunt, 2003; Mccabe et al., 2004; Taconnat et al., 2006). Another possibility is that differences in performance across the different image categories do not arise due to a distinction between perceptual versus conceptual encoding but rather due to differences in perceptual encoding and hence the type of representations formed and used in working memory. For example, less homogeneous images containing more detail may be encoded on the basis of distinguishing features rather than a more holistic representation of the image’s identity. The notion that faces, which are highly homogeneous and are recognized at the individual level, may be encoded and recognized using more holistic information has been extensively discussed in the literature (Maurer et al., 2002; Richler et al., 2008). Moreover, some have argued that aging may impact this process (Chaby et al., 2001; Obermeyer et al., 2012; but see Hildebrandt et al., 2010; Boutet et al., 2015), which might explain why aging had a greater effect on homogeneous as compared to heterogeneous stimuli.

A second limitation which must be taken into account when interpreting our findings is that comparisons with upright faces are limited by the near-ceiling effect in number of false alarms obtained for this condition, which arose from differences in speed-accuracy trade-off across conditions. Older adults seem to have adopted a strategy whereby they made fewer mistakes for the upright faces but took more time to provide an answer. It is unclear whether this tendency is driven by the social relevance of faces, or to participant artifacts produced by their knowledge that this was a study about “face recognition”. Either way, our findings underscore the importance of including measures of reaction time in studies on false recognition, especially in light of processing speed theories of aging (e.g., see Salthouse, 1996 for general cognitive impairments and Rousselet et al., 2010 for upright faces). Our findings that visual ability and reaction time were not correlated suggest that these two factors may be independent. We note that results derived from false alarm efficiency, which takes into account speed-accuracy trade-offs, were not contaminated by this ceiling effect.

## Conclusion

Our results suggest that perceptual similarity can contribute to age-related differences in false recognition. Moreover, age-related perceptual decline is related to elevated false recognition for perceptually similar but not dissimilar items. Despite wearing up-to-date corrective lenses, our participants still displayed significant impairments in basic vision as compared to the younger group, suggesting that visual ability should be measured when neuropsychological tests that rely on memory are used to measure the integrity of cognitive systems. We also encourage seniors to wear corrective lenses during social interactions to avoid exacerbating difficulties with face recognition. Finally, more research is needed to clarify whether a decline in pattern separation, or a reliance on gist, provides the best interpretative framework for understanding the contribution of perceptual factors to false recognition.

## Ethics Statement

This study was carried out in accordance with the recommendations of the Canadian Tri-Council Policy Statement for Ethical Conduct for Research Involving Humans with written informed consent from all subjects. All subjects gave written informed consent in accordance with the Declaration of Helsinki. The protocol was approved by the Office of Research Ethics and Integrity of the University of Ottawa.

## Author Contributions

All authors contributed to the genesis, data collection, data analysis, and interpretation of the results. IB wrote most of the manuscript. CC edited the manuscript. KD, FC, and OB participated in data collection and wrote parts of the draft of the manuscript. KD and FC participated in data analyses.

## Conflict of Interest Statement

The authors declare that the research was conducted in the absence of any commercial or financial relationships that could be construed as a potential conflict of interest.

## Footnotes

1 We remind the reader that near-ceiling performance with upright faces was due to a speed-accuracy trade-off, which justified the addition of false alarm efficiency as a dependent variable in our analyses.

## References

[B1] Arndt J. Hirshman E. (1998). True and false recognition in minerva2: explanations from a global matching perspective. *J. Mem. Lang.* 39 371–391. 10.1006/jmla.1998.2581

[B2] Bach M. (1996). The freiburg visual acuity test. automatic measurement of visual acuity. *Optom. Vis. Sci.* 73 49–53. 10.1097/00006324-199601000-00008 8867682

[B3] Baddeley A. D. Emslie H. Nimmo-Smith I. (1994). *Doors and People: A Test of Visual and Verbal Recall and Recognition.* Bury St. Edmunds: Thames Valley Test Company.

[B4] Bastin C. Van der Linden M. (2003). The contribution of recollection and familiarity to recognition memory: a study of the effects of test format and aging. *Neuropsychology* 17 14–24. 10.1037//0894-4105.17.1.14 12597069

[B5] Benton A. L. Sivan A. B. Hamsher K. Varney N. R. Spreen O. (1994). *Contributions to Neuropsychological Assessment.* New York, NY: Oxford University Press.

[B6] Berkman L. F. Glass T. Brissette I. Seeman T. E. (2000). From social integration to health: durkheim in the new millennium. *Soc. Sci. Med.* 51 843–857. 10.1016/s0277-9536(00)00065-4 10972429

[B7] Berkman L. F. Syme S. L. (1979). Social networks, host resistance, and mortality: a nine-year follow-up study of alameda county residents. *Am. J. Epidemiol.* 109 186–204. 10.1093/oxfordjournals.aje.a112674 425958

[B8] Boutet I. Faubert J. (2006). Recognition of faces and complex objects in younger and older adults. *Mem. Cogn.* 34 854–864. 10.3758/bf03193432 17063916

[B9] Boutet I. Meinhardt-Injac B. (2018). Age differences in face processing: the role of perceptual degradation and holistic processing. *J. Gerontol. Ser. B* 10.1093/geronb/gbx172 [Epub ahead of print]. 29373754

[B10] Boutet I. Shah D. K. Collin C. Berti S. Persike M. Meinhardt-Injac B. (2019). Effets du vieillissement sur les potentiels évoquées par les visages et les objets. *Paper Presented at the 41th Annual Meeting of the Société Québecoise pour la Recherche en Psychologie*, Canada.

[B11] Boutet I. Taler V. Collin C. A. (2015). On the particular vulnerability of face recognition to aging: a review of three hypotheses. *Front. Psychol.* 6:1139. 10.3389/fpsyg.2015.01139 26347670 PMC4543816

[B12] Brainerd C. J. Reyna V. F. (2002). Fuzzy-trace theory and false memory. *Curr. Dir. Psychol. Sci.* 11 164–169. 10.1111/1467-8721.00192

[B13] Brainerd C. J. Reyna V. F. Kneer R. (1995). False-recognition reversal: when similarity is distinctive. *J. Mem. Lang.* 34 157–185. 10.1006/jmla.1995.1008

[B14] Budson A. E. Michalska K. J. Sullivan A. L. Rentz D. M. Daffner K. R. Schacter D. L. (2003). False recognition in alzheimer disease: evidence from categorized pictures. *Cogn. Behav. Neurol.* 16 16–27. 10.1097/00146965-200303000-00003 14764998

[B15] Burnside K. Hope C. Gill E. Morcom A. M. (2017). Effects of perceptual similarity but not semantic association on false recognition in aging. *PeerJ* 5:e4184. 10.7717/peerj.4184 29302398 PMC5742526

[B16] Burton A. M. White D. McNeill A. (2010). The glasgow face matching test. *Behav. Res. Methods* 42 286–291. 10.3758/BRM.42.1.286 20160307

[B17] Chaby L. Jemel B. George N. Renault B. Fiori N. (2001). An ERP study of famous face incongruity detection in middle age. *Brain Cogn.* 45 357–377. 10.1006/brcg.2000.1272 11305879

[B18] Chaby L. Narme P. (2009). La reconnaissance des visages et de leurs expressions faciales au cours du vieillissement normal et dans les pathologies neurodégénératives. *Psychol. Neuropsychiatr. Vieil.* 7 31–42. 10.1684/pnv.2008.0154 19251570

[B19] Chouinard P. A. Morrissey B. F. Köhler S. Goodale M. A. (2008). Repetition suppression in occipital–temporal visual areas is modulated by physical rather than semantic features of objects. *NeuroImage* 41 130–144. 10.1016/j.neuroimage.2008.02.011 18375148

[B20] Cohen S. (2004). Social relationships and susceptibility to the common cold. *Am. Psychol.* 59 676–684. 10.1037/0003-066X.59.8.676 15554821

[B21] Cronin-Golomb A. Gilmore G. C. Neargarder S. Morrison S. R. Laudate T. M. (2007). Enhanced stimulus strength improves visual cognition in aging and alzheimers disease. *Cortex* 43 952–966. 10.1016/s0010-9452(08)70693-2 17941352

[B22] Davidson P. Vidjen P. Trincao-Batra S. Collin C. (2018). Older adults’ lure discrimination difficulties on the mnemonic similarity test are significantly correlated with their visual perception. *J. Gerontol. Psychol. Sci.* 10.1093/geronb/gby130 [Epub ahead of print]. 30407604

[B23] Delis D. C. Kramer J. H. Kaplan E. Ober B. A. (1987). *CVLT, California Verbal Learning Test: Adult Version: Manual.* San Antonio: Psychological Corporation.

[B24] Dupuis K. Pichora-Fuller M. K. Chasteen A. L. Marchuk V. Singh G. Smith S. L. (2014). Effects of hearing and vision impairments on the montreal cognitive assessment. *Aging Neuropsychol. Cogn.* 22 413–437. 10.1080/13825585.2014.968084 25325767

[B25] Evenhuis H. M. (1996). Further evaluation of the dementia questionnaire for persons with mental retardation (DMR). *J. Intellect. Disabil. Res.* 40 369–373. 10.1111/j.1365-2788.1996.tb00642.x 8884592

[B26] Faul F. Erdfelder E. Lang A.-G. Buchner A. (2007). G^∗^ Power 3: a flexible statistical power analysis program for the social, behavioral, and biomedical sciences. *Behav. Res. Methods* 39 175–191. 10.3758/bf03193146 17695343

[B27] Flicker C. Ferris S. H. Crook T. Bartus R. T. (1990). Impaired facial recognition memory in aging and dementia. *Alzheimer Dis. Assoc. Disord.* 4 43–54. 10.1097/00002093-199040100-00005 2317337

[B28] Gauthier I. Williams P. Tarr M. J. Tanaka J. (1998). Training “greeble” experts: a framework for studying expert objects recognition processes. *Vis. Res.* 38 2401–2428. 10.1016/s0042-6989(97)00442-2 9798007

[B29] Gold J. Bennett P. J. Sekuler A. B. (1999). Identification of band-pass filtered letters and faces by human and ideal observers. *Vision Res.* 39 3537–3560. 10.1016/s0042-6989(99)00080-2 10746125

[B30] Hildebrandt A. Sommer W. Herzmann G. Wilhelm O. (2010). Structural invariance and age-related performance differences in face cognition. *Psychol. Aging* 25 794–810. 10.1037/a0019774 20822255

[B31] Hunt R. R. (2003). Two contributions of distinctive processing to accurate memory. *J. Mem. Lang.* 48 811–825. 10.1016/s0749-596x(03)00018-4

[B32] Kouststaal W. Reddy C. Jackson E. M. Prince S. Cendan D. L. Schacter D. L. (2003). False recognition of abstract versus common objects in older and younger adults: testing the semantic categorization account. *J. Exp. Psychol. Learn. Mem. Cogn.* 29 499–510. 10.1037/0278-7393.29.4.499 12924853

[B33] Koutstaal W. (2003). Older adults encode—but do not always use perceptual details. *Psychol. Sci.* 14 189–193. 10.1111/1467-9280.01441 12661684

[B34] Koutstaal W. Schacter D. L. (1997). Gist-based false recognition of pictures in older and younger adults. *J. Mem. Lang.* 37 555–583. 10.1006/jmla.1997.2529 10403710

[B35] Koutstaal W. Schacter D. L. Brenner C. (2001a). Dual task demands and gist-based false recognition of pictures in younger and older adults. *J. Mem. Lang.* 44 399–426. 10.1006/jmla.2000.2734

[B36] Koutstaal W. Wagner A. Rotte M. Maril A. Buckner R. Schacter D. (2001b). Perceptual specificity in visual object priming: functional magnetic resonance imaging evidence for a laterality difference in fusiform cortex. *Neuropsychologia* 39 184–199. 10.1016/s0028-3932(00)00087-7 11163375

[B37] Koutstaal W. Schacter D. L. Galluccio L. Stofer K. A. (1999). Reducing gist-based false recognition in older adults: encoding and retrieval manipulations. *Psychol. Aging* 14 220–237. 10.1037//0882-7974.14.2.220 10403710

[B38] Lamont A. C. Stewart-Williams S. Podd J. (2005). Face recognition and aging: effects of target age and memory load. *Mem. Cogn.* 33 1017–1024. 10.3758/bf03193209 16496722

[B39] Lawton M. Kasten M. May M. T. Mollenhauer B. Schaumburg M. Liepelt-Scarfone I. (2016). Validation of conversion between mini-mental state examination and montreal cognitive assessment. *Mov. Disord.* 31 593–596. 10.1002/mds.26498 26861697 PMC4864892

[B40] Lieberman H. R. Pentland A. P. (1982). Microcomputer-based estimation of psychophysical thresholds: the best PEST. *Behav. Res. Methods Instrum.* 14 21–25. 10.3758/BF03202110

[B41] Lövdén M. (2003). The episodic memory and inhibition accounts of age-related increases in false memories: a consistency check^∗^1. *J. Mem. Lang.* 49 268–283. 10.1016/s0749-596x(03)00069-x

[B42] Ly M. Murray E. Yassa M. A. (2013). Perceptual versus conceptual interference and pattern separation of verbal stimuli in young and older adults. *Hippocampus* 23 425–430. 10.1002/hipo.22110 23505005 PMC3968906

[B43] Macmillan N. A. Creelman C. D. (2005). *Detection Theory: A User’s Guide*, 2nd Edn. New York, NY: Psychological Press.

[B44] Maurer D. Grand R. L. Mondloch C. J. (2002). The many faces of configural processing. *Trends Cogn. Sci.* 6 255–260. 10.1016/s1364-6613(02)01903-4 12039607

[B45] Mccabe D. P. Presmanes A. G. Robertson C. L. Smith A. D. (2004). Item-specific processing reduces false memories. *Psychon. Bull. Rev.* 11 1074–1079. 10.3758/bf03196739 15875978

[B46] McKone E. Kanwisher N. Duchaine B. C. (2006). Can generic expertise explain special processing for faces? *Trends Cogn. Sci.* 11 8–15. 10.1016/j.tics.2006.11.002 17129746

[B47] Meinhardt-Injac B. Boutet I. Persike M. Meinhardt G. Imhof M. (2017). From development to aging: holistic face perception in children, younger and older adults. *Cognition* 158 134–146. 10.1016/j.cognition.2016.10.020 27835784

[B48] Meinhardt-Injac B. Persike M. Meinhardt G. (2014a). Holistic face perception in young and older adults: effects of feedback and attentional demand. *Front. Aging Neurosci.* 6:291. 10.3389/fnagi.2014.00291 25386138 PMC4208490

[B49] Meinhardt-Injac B. Persike M. Meinhardt G. (2014b). Holistic processing and reliance on global viewing strategies in older adults’ face perception. *Acta Psychol.* 151 155–163. 10.1016/j.actpsy.2014.06.001 24977938

[B50] Monge Z. A. Madden D. J. (2016). Linking cognitive and visual perceptual decline in healthy aging: the information degradation hypothesis. *Neurosci. Biobehav. Rev.* 69 166–173. 10.1016/j.neubiorev.2016.07.031 27484869 PMC5030166

[B51] Näsänen R. (1999). Spatial frequency bandwidth used in the recognition of facial images. *Vision Res.* 39 3824–3833. 10.1016/S0042-6989(99)00096-6 10748918

[B52] Neargarder S. A. Stone E. R. Cronin-Golomb A. Oross S. III. (2003). The impact of acuity on performance of four clinical measures of contrast sensitivity in Alzheimer’s disease. *Psychol. Sci. Soc. Sci.* 58 54–62. 10.1093/geronb/58.1.P54 12496302

[B53] Norman K. A. O’Reilly R. C. (2003). Modeling hippocampal and neocortical contributions to recognition memory: a complementary-learning-systems approach. *Psychol. Rev.* 110 611–646. 10.1037/0033-295X.110.4.611 14599236

[B54] Norton D. McBain R. Chen Y. (2009). Reduced ability to detect facial configuration in middle-aged and elderly individuals: associations with spatiotemporal visual processing. *Psychol. Sci. Soc. Sci.* 64B, 328–334. 10.1093/geronb/gbp008 19255087 PMC2905137

[B55] Obermeyer S. Kolling T. Schaich A. Knopf M. (2012). Differences between old and young adults’ ability to recognize human faces underlie processing of horizontal information. *Front. Aging Neurosci.* 4:3 10.3389/fnagi.2012.00003 PMC3332157 22536184

[B56] Owsley C. Sekuler R. Boldt C. (1981). Aging and low-contrast vision: face perception. *Invest. Ophthalmol. Vis. Sci.* 21 362–365. 7251315

[B57] Pidgeon L. M. Morcom A. M. (2014). Age-related increases in false recognition: the role of perceptual and conceptual similarity. *Front. Aging Neurosci.* 6:283. 10.3389/fnagi.2014.00283 25368576 PMC4201095

[B58] Pidgeon L. M. Morcom A. M. (2016). Cortical pattern separation and item-specific memory encoding. *Neuropsychologia* 85 256–271. 10.1016/j.neuropsychologia.2016.03.026 27018483

[B59] Ramon M. Rossion B. (2012). Hemisphere-dependent holistic processing of familiar faces. *Brain Cogn.* 78 7–13. 10.1016/j.bandc.2011.10.009 22099150

[B60] Rankin J. L. Kausler D. H. (1979). Adult age differences in false recognitions. *J. Gerontol.* 34 58–65. 10.1093/geronj/34.1.58 759493

[B61] Rémy P. Taconnat L. Isingrini M. (2008). Effects of aging and attention-demanding tasks on false recognition induced by photographs: differences between conceptually and perceptually modified lures. *Exp. Aging Res.* 34 220–231. 10.1080/03610730802070118 18568980

[B62] Reyna V. F. Brainerd C. J. (1991). Fuzzy-trace theory and framing effects in choice: gist extraction, truncation, and conversion. *J. Behav. Decis. Making* 4 249–262. 10.1002/bdm.3960040403

[B63] Richler J. J. Gauthier I. Wenger M. J. Palmeri T. J. (2008). Holistic processing of faces: perceptual and decisional components. *J. Exp. Psychol. Learn. Mem. Cogn.* 34 328–342. 10.1037/0278-7393.34.2.328 18315409

[B64] Rosenthal R. Rosnow R. L. (1985). *Contrast Analysis: Focused Comparisons in the Analysis of Variance.* Cambridge: Cambridge University Press.

[B65] Rousselet G. Husk J. Pernet C. Gaspar C. Bennett P. Sekuler A. (2010). Age-related delay in information accrual for faces: evidence from a parametric, single-trial EEG approach. *J. Vis.* 9 545–545. 10.1167/9.8.545 19740414 PMC2746225

[B66] Rousselet G. A. Husk J. S. Bennett P. J. Sekuler A. B. (2008). Time course and robustness of ERP object and face differences. *J. Vis.* 8 3.–3.18. 10.1167/8.12.3 18831616

[B67] Salthouse T. A. (1996). The processing-speed theory of adult age differences in cognition. *Psychol. Rev.* 103 403–428. 10.1037/0033-295X.103.3.403 8759042

[B68] Schacter D. L. Koutstaal W. Johnson M. K. Gross M. S. Angell K. E. (1997a). False recollection induced by photographs: a comparison of older and younger adults. *Psychol. Aging* 12 203–215. 10.1037/0882-7974.12.2.203 9189980

[B69] Schacter D. L. Norman K. A. Koutstaal W. (1997b). “The recovered memories debate: A cognitive neuroscience perspective,” in *Debates in Psychology. Recovered Memories and False Memories*, ed. Conway M. A. (New York, NY: Oxford University Press), 63–99. 10.1093/med:psych/9780198523864.003.0004

[B70] Schacter D. L. Verfaellie M. Anes M. D. (1997c). Illusory memories in amnesic patients: conceptual and perceptual false recognition. *Neuropsychology* 11 331–342. 10.1037/0894-4105.11.3.331 9223138

[B71] Schiller J. S. Peregoy J. A. (2012). *Provisional Report: Summary health statistics for U.S. adults: National Health Interview Survey*, 2011. Hyattsville: National Center for Health Statistics. 25116400

[B72] Searcy J. H. Bartlett J. C. Memon A. (1999). Age differences in accuracy and choosing in eyewitness identification and face recognition. *Mem. Cogn.* 27 538–552. 10.3758/BF03211547 10355242

[B73] Sekuler A. B. Gaspar C. M. Gold J. M. Bennett P. J. (2004). Inversion leads to quantitative, not qualitative, changes in face processing. *Curr. Biol.* 14 391–396. 10.1016/j.cub.2004.02.028 15028214

[B74] Shiffrin R. M. Huber D. E. Marinelli K. (1995). Effects of category length and strength on familiarity in recognition. *J. Exp. Psychol. Learn. Mem. Cogn.* 21 267–287. 10.1037/0278-7393.21.2.267 7738500

[B75] Smith A. D. Winograd E. (1978). Adult age differences in remembering faces. *Dev. Psychol.* 14 443–444. 10.1037/0012-1649.14.4.443

[B76] Sommers M. S. Huff L. M. (2003). The effects of age and dementia of the alzheimer’s type on phonological false memories. *Psychol. Aging* 18 791–806. 10.1037/0882-7974.18.4.791 14692865

[B77] Spear P. D. (1993). Neural bases of visual deficits during aging. *Vision Res.* 33 2589–2609. 10.1016/0042-6989(93)90218-L 8296455

[B78] Stahl C. Henze L. Aust F. (2016). *False Memory for Perceptually Similar But Conceptually Distinct Line Drawings.* Cologne: University of Cologne.

[B79] Stark S. M. Yassa M. A. Lacy J. W. Stark C. E. (2013). A task to assess behavioral pattern separation (BPS) in humans: data from healthy aging and mild cognitive impairment. *Neuropsychologia* 51 2442–2449. 10.1016/j.neuropsychologia.2012.12.014 23313292 PMC3675184

[B80] Taconnat L. Clarys D. Vanneste S. Isingrini M. (2006). Effects of distinctive encoding on false recognition, discrimination and decision criteria in young and elderly adults. *Eur. J. Cogn. Psychol.* 18 708–729. 10.1080/09541440500285585

[B81] Taconnat L. Rémy P. (2006). Les faux souvenirs dans le vieillissement normal: données empiriques et modèles théoriques. *L’année Psychologique* 106 457–486. 10.4074/S0003503306003071

[B82] Tanaka J. Gauthier I. (1997). Expertise in object and face recognition. *Psychol. Learn. Motiv. Perc. Learn.* 36 83–125. 10.1016/s0079-7421(08)60282-0

[B83] Thomas A. K. Sommers M. S. (2005). Attention to item-specific processing eliminates age effects in false memory. *J. Mem. Lang.* 52 71–86. 10.1016/j.jml.2004.08.001

[B84] Toner C. K. Pirogovsky E. Kirwan C. B. Gilbert P. E. (2009). Visual object pattern separation deficits in nondemented older adults. *Learn. Mem.* 16 338–342. 10.1101/lm.1315109 19403797

[B85] Townsend J. T. Ashby F. G. (1978). “Methods of modeling capacity in simple processing systems,” in *Cognitive Theory* Vol. 3 eds Castellan J. Restle F. (Hillsdale, N.J: Erlbaum), 200–239.

[B86] Trahan D. E. Larrabee G. J. Levin H. S. (1986). Age-related differences in recognition memory for pictures. *Exp. Aging Res.* 12 147–150. 10.1080/03610738608259452 3830233

[B87] Tun P. A. Wingfield A. Rosen M. J. Blanchard L. (1998). Response latencies for false memories: gist-based processes in normal aging. *Psychol. Aging* 13 230–241. 10.1037/0882-7974.13.2.230 9640584

[B88] Umberson D. Williams K. Powers D. A. Liu H. Needham B. (2006). You make me sick: marital quality and health over the life course. *J. Health Soc. Behav.* 47 1–16. 10.1177/002214650604700101 16583772 PMC3149975

[B89] Vandierendonck A. (2017). A comparison of methods to combine speed and accuracy measures of performance: a rejoinder on the binning procedure. *Behav. Res. Methods* 49 653–673. 10.3758/s13427-016-0721-5 26944576

[B90] Wang F. Ford D. Tielsch J. M. Quigley H. A. Whelton P. K. (1994). Undetected eye disease in a primary care clinic population. *Arch. Intern. Med.* 154:1821. 10.1001/archinte.1994.00420160054007 8053749

[B91] Wang Y. Zhou Y. Ma Y. Leventhal A. G. (2005). Degradation of signal timing in cortical areas V1 and V2 of senescent monkeys. *Cereb. Cortex* 15 403–408. 10.1093/cercor/bhh143 15749984

[B92] Weinstein Y. Shanks D. R. (2008). Perceptual representations in false recognition and priming of pictures. *Mem. Cogn.* 36 1415–1428. 10.3758/mc.36.8.1415 19015501

[B93] Wilson I. A. Gallagher M. Eichenbaum H. Tanila H. (2006). Neurocognitive aging: prior memories hinder new hippocampal encoding. *Trends Neurosci.* 29 662–670. 10.1016/j.tins.2006.10.002 17046075 PMC2614702

[B94] Yassa M. A. Lacy J. W. Stark S. M. Albert M. S. Gallagher M. Stark C. E. (2011a). Pattern separation deficits associated with increased hippocampal CA3 and dentate gyrus activity in nondemented older adults. *Hippocampus* 21 968–979. 10.1002/hipo.20808 20865732 PMC3010452

[B95] Yassa M. A. Mattfeld A. T. Stark S. M. Stark C. E. (2011b). Age-related memory deficits linked to circuit-specific disruptions in the hippocampus. *Proc. Natl. Acad. Sci.* 108 8873–8878. 10.1073/pnas.1101567108 21555581 PMC3102362

[B96] Zeki S. M. (1978). Uniformity and diversity of structure and function in rhesus monkey prestriate visual cortex. *J. Physiol.* 277 273–290. 10.1113/jphysiol.1978.sp012272 418176 PMC1282389

